# Time-course gene expression profiling data of *Triticum aestivum* treated by supercritical CO_2_ garlic extract encapsulated in nanoscale liposomes

**DOI:** 10.1016/j.dib.2022.108287

**Published:** 2022-05-17

**Authors:** Barbara Kutasy, Kincső Decsi, Márta Kiniczky, Géza Hegedűs, Eszter Virág

**Affiliations:** a Department of Plant Physiology and Plant Ecology, the Hungarian University of Agriculture and Life Sciences, Georgikon Campus Keszthely, 8360 Keszthely, Festetics Gy. u. 7., Hungary; b Research Institute for Medicinal Plants and Herbs Ltd. 2011 Budakalász, Lupaszigeti út 4., Hungary; c EduCoMat Ltd., 830 Keszthely, Iskola u. 12/A., Hungary; d Department of Molecular Biotechnology and Microbiology, Institute of Biotechnology, Faculty of Science and Technology, University of Debrecen, 4132 Debrecen, Egyetem tér 1., Hungary

**Keywords:** Illumina sequencing, *Allium sativum*, Nanotechnology, Whole-genome transcriptional profiling, Defense response, Biostimulant

## Abstract

The biostimulant phytochemicals as alternatives to synthetic chemicals are gaining ground in sustainable agricultural production nowadays. The medicinal herb, garlic *(Allium sativum)* has a spectacular therapeutic reputation due to its antimicrobial properties. The effectiveness of supercritical carbon dioxide (SC-CO_2_) extraction of *A. sativum* could help preserve bioactive compounds and be used as a biostimulant agent. The SC-CO_2_ garlic was formulated in liposomes and used as a nanoscale drug delivery system to reach better efficiency of penetration and translocation. The SC-CO_2_ garlic extracts were used in *Triticum aestivum* time-course experiments to monitor conditioning effects such as improving crop quality and priming its defense responses against different pathogens. Fresh leaves were collected after SC-CO_2_ garlic exposure at 15 min, 24, and 48 hours for QuantSeq 3′ mRNA sequencing at Illumina NextSeq 550 platform. RNA quantification datasets are presented. Raw data such as Illumina 85bp single-end read sequences and reconstructed transcripts were deposited in the NCBI SRA and TSA databases under the BioProject PRJNA808851. Functional annotation of transcripts and time-course expression data are presented here to support gene expression analysis experiments.

## Specifications Table

**Table:** 

Subject	Plant Science: Plant Physiology
Specific subject area	The effect of liposome-formulated supercritical carbon dioxide (SC-CO_2_) garlic extract (named *‘garlic-lipo’*) with high antimicrobial potential was examined to stimulate defense mechanisms in wheat (*Triticum aestivum)*.
Type of data	TableDatabase recordFigure
How the data were acquired	The foliar treatment of ‘garlic-lipo’ was performed in a greenhouse experiment using *T. aestivum* plants. Dose corresponding to 240 g/ha was set and in four time points were the samples collected upon exposure. Total RNA of leaves tissues of four investigated samples were used to prepare QuantSeq 3′ mRNA NGS libraries for NextSeq550 sequencing. The final output of single-end libraries were 14-26 M x 85 bases long. Quality control was performed with FastQC that was followed by preprocessing of raw reads. Using *de novo* assembly of short, preprocessed reads, a combined transcript dataset was obtained and RNA quantification of each sample were performed.
Data format	RawAnalyzedFiltered
Description of data collection	*T. aestivum* plants were sprayed with 240g/ha ‘garlic-lipo’ at the BBCH12 stage under greenhouse conditions. Sample collection: 30 mg of fresh leaves were collected before treatment, as control (0) and 15 min, 24 and 48 hours after treatment. Plant materials were stored in RNA Shield at -25°C until RNA sequencing.
Data source location	EduCoMat Ltd Keszthely Hungary
Data accessibility	The bio project and raw reads are available in National Center for Biotechnology Information database under the accessions:Repository name: *Triticum aestivum* cultivar: Cellule (bread wheat)Data identification number: PRJNA808851Direct link to datasets: https://www.ncbi.nlm.nih.gov/bioproject/PRJNA808851Repository name: CountTable and AnnotationTable as Supplementary 1-2Data identification number (DOI): 10.17632/p66v4yxbtp.1Direct link to datasets: https://data.mendeley.com/datasets/p66v4yxbtp/1Repository name: TimeCourseTable as Supplementary 3Data identification number (DOI): 10.17632/xvvscxpz6w.1Direct link to datasets: https://data.mendeley.com/datasets/xvvscxpz6w/1

## Value of the Data

• Currently, there is a clear need to enhance the sustainability of agricultural production systems with efficient and environmentally safe methods. To research the bioactivity of garlic extract as a biostimulant is useful because it may improve crop quality with the potential in priming and triggering defense responses against pathogens.
• Liposome-assisted drug delivery is a promising technology to increase the effectiveness of the active agents. To date, no data of SC-CO_2_ garlic extract encapsulated in nanoscale liposomes is reported. This is the first report on biological data investigated with SC-CO_2_ garlic formulated in liposomes as ‘garlic-lipo’.
• The use of garlic extracts is well-reported in vegetables but not in arable crops. These data may contribute to applied research of effective plant protection of *T. aestivum* that may use in organic farming as well.
• Moreover, Illumina GEx sequencing system was used to perform comprehensive gene expression profiling. These data contribute to the molecular genetic background of the changes of plant physiological processes as a function of time in the case of wheat.

## 1 Data Description

Using plant-based extracts, such as garlic extract as the plant biostimulant (PB) is effective to enhance the sustainability of crop protection and may increase defense responses to abiotic and biotic stresses [[1], [2], [3]–4]. Supercritical CO_2_ extraction technique and nanoscale drug delivery system (liposomes composed of plant-derived lipids) have been shown to more efficient penetration and translocation than many liquid organic solvents [[5], [6]–7]. We performed a liposome formulation of organic SC-CO_2_ garlic extract by using sunflower lecithin (‘garlic-lipo’).

**Fig. 1:**
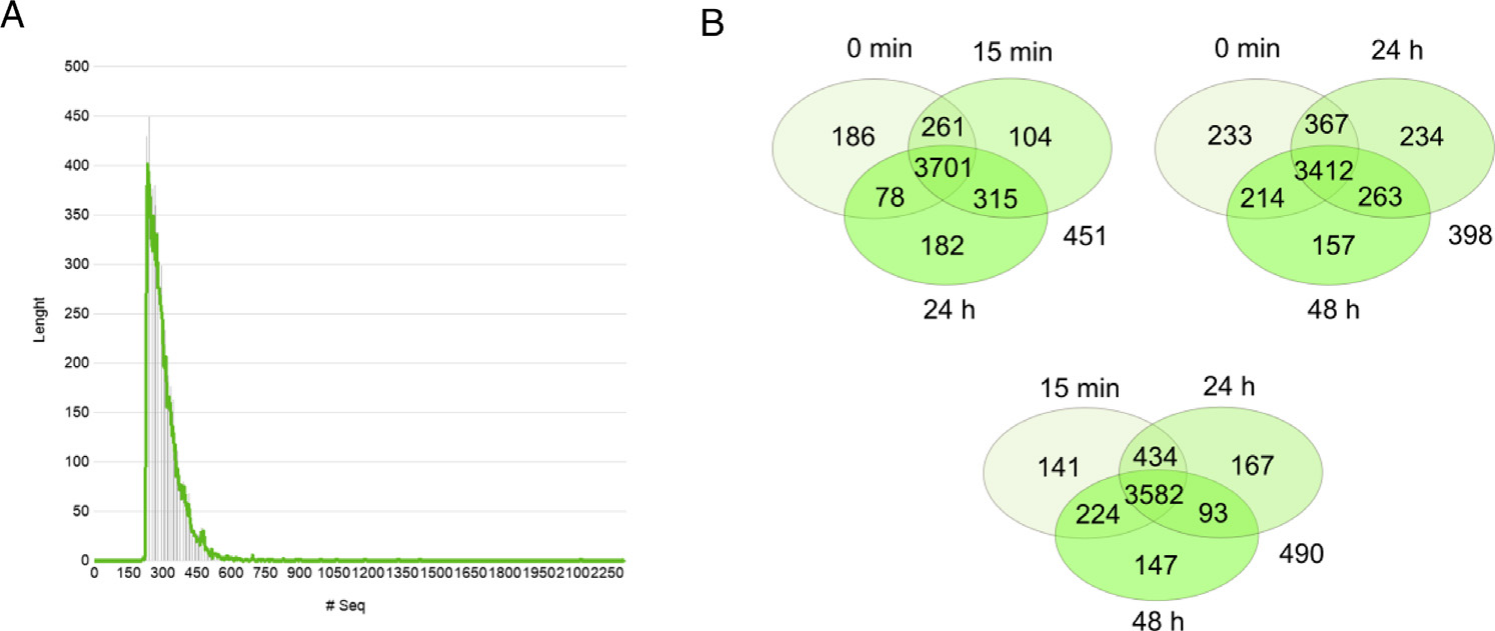
Number of sequences with length of TSA data (A); Venn diagrams of transcript distributions, for better understanding we present in 3 Venn diagrams the numerical data of four samples. Numbers outside the sets are numbers of transcripts without abundances (B).

**Fig. 2:**
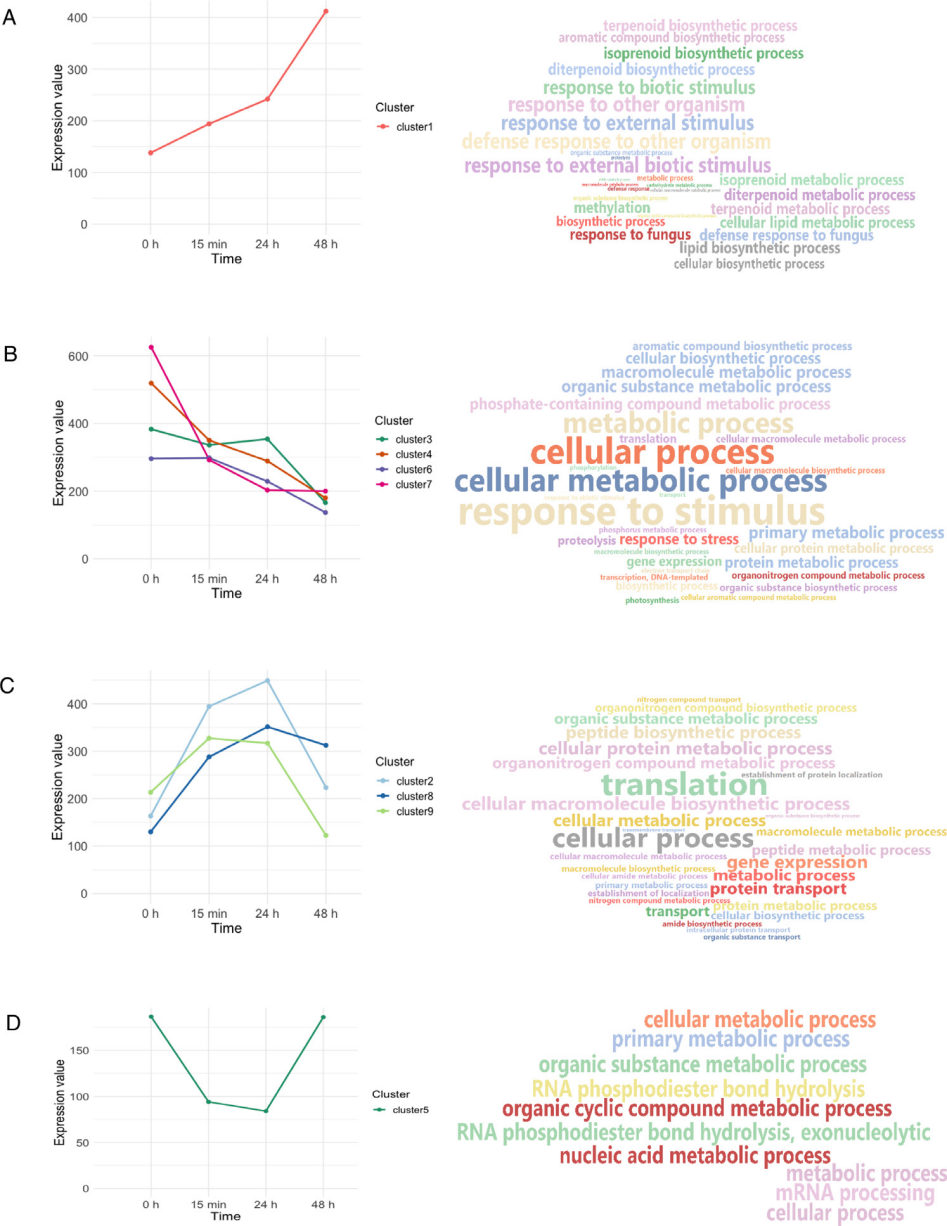
Time-course expression alterations. Graph showing the median level expression (significance level 0.05; R-squared cutoff 0.7) of each cluster of genes across times. WordClouds represent Gene Ontology (GO) term (biological process) summing annotated genes of each group, the font size depends on the sequence count. Genes with significantly different expression levels were classified into 9 clusters according to the dynamics of change: expression level gradually was increased (A), gradually decreased (B), initially increased and then reduced (C) initially decreased and then returned to its original level (D).

Greenhouse experiments of wheat were set to investigate the plant conditioning effect of ‘garlic-lipo’. The time-course experiment of wheat treated with an amount equivalent to 240g/ha ‘garlic-lipo’ and monitored in 0 min, 15 min, 24, and 48 hours. QuantSeq 3′ mRNA sequencing for RNA quantification [8] was performed with four samples collected in these time points. Sequencing was performed at Illumina NextSeq550 platform to produce 85bp long reads. Preprocessed sequence reads are deposited in the NCBI Sequence Read Archive (SRA) database under the Bioproject PRJNA808851 with accession numbers SRR18107544, SRR18107543, SRR18107542 and SRR18107541. Using SRA datasets *de novo* assembly was performed to reconstruct transcripts that are deposited in Transcriptome Shotgun Assembly (TSA) database at DBJ/EMBL/GenBank under the accession GJUY00000000. The version described in this paper is the first version GJUY01000000. The deposited transcripts length distribution is summarized (Fig. 1A). Read counts were specified in the CountTable (Supplementary 1) that contain the total transcript abundances per each sample, distributions of unique and common transcripts are visualized using Venn diagrams (Fig. 1B). Using TSA and CountTable data, time-course expression analysis was performed (Supplementary 3). The time-course expression analysis resulted in 9 clusters changing in time (Fig. 2). Functional annotation of these 9 clusters is detailed (Supplementary 2). The number of transcripts and annotation statistics are summarized (Table 1.) and the filtered top 30 Gene Ontology (GO) categories (biological process) are visualized (Fig. 2).

**Table 1:** Numerical data of time-course expression analysis that was resulted in 9 clusters changing in time. Data shows the number of transcripts and annotation statistics of each cluster.

Cluster	Total transcripts	With Blast Hits	With GO Mapping	With GO Annotation
1	201	100	77	56
2	90	44	34	14
3	212	123	78	47
4	265	163	122	82
5	18	8	6	3
6	119	65	50	36
7	55	38	31	23
8	33	13	12	8
9	40	20	12	10

## 2 Experimental Design, Materials and Methods

### 2.1 Plant materials

CO_2_ garlic extract was purchased from Flavex Naturextrakte GmbH (Nordstraße 7, D-66780 Rehlingen). Seeds of *T. aestivum* cultivar ‘Cellule’ were germinated in BD Avantgarde.Line incubator (Binder GmbH, Tuttlingen, Germany) at 25°C/15°C, 16 h photoperiod and were planted in pots than were growing under controlled greenhouse condition. Plants were sprayed with ‘garlic-lipo’ 240g/ha using automatic spraying chamber (Euro Pulvé, Aspach, France) at BBCH12 stage. Fresh leaves were collected before treatment, as control (0) and 15 min, 24 and 48 h after treatment. Samples were stored in RNA Shield (Zymo research, Irvine, CA, US) at -25°C until RNA-sequencing and analysis.

### 2.2 Sequencing and data processing

The Illumina library preparation, sequencing, pre-processing, transcriptome assembly and determination of transcript abundances for gene level quantification were performed as described in our previous publication by Hegedűs [9].

### 2.3 Time-course expression analysis

To the detection of genomic features with significant temporal expression changes and significant differences between experimental groups the software package ‘maSigPro’ (belonging to the Bioconductor project) was used. In this analysis, this software applies a two steps regression strategy to find genes that show significant expression changes over time and between experimental groups [10]. At the beginning of the analysis the number of total features was 5,287 and the differentially expressed features identified (False Discovery Rate < 0.05) was 2,856. The number of blasted genes was 584 and the number of stress-respond-associated genes were 207. Genes with significantly different expression levels were classified into 9 clusters according to the dynamics of change.

### 2.4 Functional annotation

To achieve the efficient functional annotation and GO analysis of 9 clusters OmicsBox. Biobam software was practiced as described in our previous publication by Decsi et al. [11]. The GO categories of upregulated genes were symbolized by WordCloud based on GO IDs Fisher score algorithm.

## CRediT Author Statement

**Barbara Kutasy:** Investigation, Validation, Writing – original draft preparation; Visualization; **Kincső Decsi:** Validation; **Márta Kiniczky:** Preparation of investigated material ‘garlic-lipo’; **Géza Hegedűs:** Software, Investigation; **Eszter Virág:** Conceptualization, Supervision, Writing – Original draft preparation.

## Declaration of Competing Interest

The authors declare that they have no known competing financial interests or personal relationships that could have appeared to influence the work reported in this paper.

## Data Availability

TimeCourse Table of Plant sample from SC-CO2 garlic extract treated Triticum aestivum (Original data) (Mendeley Data). Plant sample from SC-CO2 garlic extract treated Triticum aestivum (Original data) (Mendeley Data).
